# Influence of Different Device Structures on the Degradation for Trench-Gate SiC MOSFETs: Taking Avalanche Stress as an Example

**DOI:** 10.3390/ma15020457

**Published:** 2022-01-08

**Authors:** Zhaoxiang Wei, Hao Fu, Xiaowen Yan, Sheng Li, Long Zhang, Jiaxing Wei, Siyang Liu, Weifeng Sun, Weili Wu, Song Bai

**Affiliations:** 1 National ASIC System Engineering Research Center, School of Electronic Science and Engineering, Southeast University, Nanjing 210096, China; weizhaoxiang27@163.com (Z.W.); fuhaoseu@163.com (H.F.); yanxiaowen_102@163.com (X.Y.); zzulisheng@163.com (S.L.); xzzlseu_ic@126.com (L.Z.); liusy2855@163.com (S.L.); swffrog@seu.edu.cn (W.S.); 2 Nanjing Electronic Devices Institute, Nanjing 100048, China; wl-wwl@sohu.com (W.W.); songer@gmail.com (S.B.)

**Keywords:** SiC MOSFET, trench gate, different device structure, degradation, avalanche

## Abstract

The variations in the degradation of electrical characteristics resulting from different device structures for trench-gate SiC metal-oxide-semiconductor field effect transistors (MOSFETs) are investigated in this work. Two types of the most advanced commercial trench products, which are the asymmetric trench SiC MOSFET and the double-trench SiC MOSFET, are chosen as the targeted devices. The discrepant degradation trends caused by the repetitive avalanche stress are monitored. For the double-trench device, the conduction characteristic improves while the gate-drain capacitance (C_gd_) increases seriously. It is because positive charges are injected into the bottom gate oxide during the avalanche process, which are driven by the high oxide electronic field (E_ox_) and the high impact ionization rate (I.I.) there. Meanwhile, for the asymmetric trench SiC MOSFET, the I–V curve under the high gate bias condition and the C_gd_ remain relatively stable, while the trench bottom is well protected by the deep P+ well. However, it’s threshold voltage (V_th_) decreases more obviously when compared with that of the double-trench device and the inclined channel suffers from more serious stress than the vertical channel. Positive charges are more easily injected into the inclined channel. The phenomena and the corresponding mechanisms are analyzed and proved by experiments and technology computer-aided design (TCAD) simulations.

## 1. Introduction

With etching, oxidation, and other critical processing technologies becoming more and more mature, SiC metal-oxide-semiconductor field effect transistors (MOSFETs) are gradually applied to power electronic fields to replace traditional silicon power devices [1,2,3,4]. Numerous SiC MOSFET products have been pushed into the market [5,6,7]. Due to the advantages of low-specific ON resistance (R_onsp_), high power density, fast switching speed, and low switching loss, trench-gate SiC MOSFETs are more promising than planar-gate ones [8,9,10]. However, for the sidewall and the bottom of a trench gate, which suffer from higher electric fields during practical applications, trench-gate SiC MOSFETs are faced with more serious reliability issues when compared with planar-gate devices [11,12].

Infineon and Rohm have released their trench-gate SiC MOSFET products in recent years, which are state-of-art commercial trench-gate devices [13,14]. The reliability of them under different stresses, such as the surge current stress, the avalanche stress, and the short-circuit stress, has been well studied [15,16,17,18,19,20,21,22,23,24,25,26]. Most of the existing articles only report the failure or the degradation mechanism of one single trench-gate device [15,16,17,18,19,20,21,22]. Very few of them investigated the failure of both devices at the same time [23,24,25,26]. However, no one reported that the disparate device structures would result in different degradations in the device performances, even if they are both trench-gate SiC MOSFETs.

In this work, the variations in the degradation trends resulting from the structure difference between two types of trench-gate devices, which are the double-trench SiC MOSFET produced by Rohm and the asymmetric trench SiC MOSFET produced by Infineon, are investigated in detail. The avalanche stress, which applies load current (I_load_) and extremely high breakdown voltage (BV) to a device simultaneously and leads to obvious degradation or even damage, is chosen as the targeted stress. It is found that after enduring repetitive avalanche stress, the static and dynamic characteristics of the two trench-gate devices exhibit different degradation trends. With the help of Silvaco TCAD simulations, the dominant mechanism is found and proved by analyzing the physical characteristics of both the devices under avalanche state.

## 2. Device Structures and Experiment Conditions

**Figure 1:**
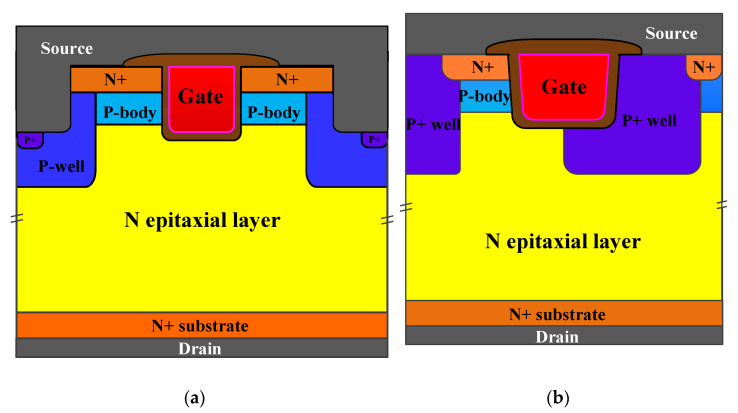
Schematic cross-sections of different trench-gate SiC MOSFETs. (**a**) The double-trench SiC MOSFET and (**b**) the asymmetric trench SiC MOSFET.

The cross-sectional schematic diagrams of the double-trench SiC MOSFET (SCT3160KL) and the asymmetric trench SiC MOSFET (IMW120R140M1H) studied in this work are shown in Figure 1a,b. Their rated BV is 1200 V, as listed in Table 1. The rated ON-state resistance (R_on_) and the DC drain current (I_D_) of the double-trench SiC MOSFET were 160 mΩ and 18 A, while the R_on_ and the I_D_ of the asymmetric trench SiC MOSFET were 140 mΩ and 19 A. They share similar conduction and blocking characteristics.

The double-trench SiC MOSFET has not only a gate trench to form the two vertical channels but also a source trench. The length and depth of each trench were both 1 μm. The P-well in the source trench can reduce the bottom electric field of the trench gate. For an asymmetric trench SiC MOSFET, there is only one channel in a single cell. The right corner of the gate is surrounded by the highly doped deep P+ well, which extends to the bottom of the trench gate, protecting the left trench corner where current flows. As shown in Figure 1b, different from the double-trench device, the left trench sidewall of the asymmetric device was parallel to the (11–20) crystal plane. Since the N epitaxial layer is homoepitaxially grown on a 4° off-axis 4H–SiC (0001) substrate, the channel of the asymmetric trench SiC MOSFET is inclined [27]. At the same time, the (11–20) crystal plane provides twice the channel mobility of other crystal planes, which improves the current density of the asymmetric trench SiC MOSFET [27,28].

The cell pitch of the double-trench SiC MOSFET was 3.6 μm, while that of the asymmetric one was 3.2 μm. The doping concentrations of the P-body and N-drift region of the double-trench device were set to be 1 × 10^17^ cm^−3^ and 8 × 10^15^ cm^−3^, while those of the asymmetric one were 1 × 10^17^ cm^−3^ and 1 × 10^16^ cm^−3^, respectively. All the cell dimensions and the doping concentrations were modified based on the real device structures and the measured characteristics. The simulations performed in this paper were based on the device structures in Figure 1.

**Figure 2:**
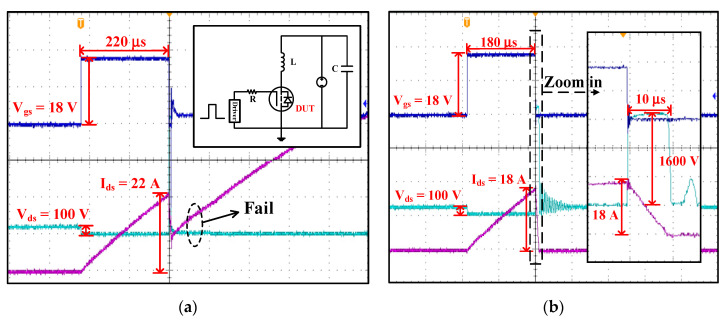
The avalanche waveforms of the asymmetric trench SiC MOSFET. (**a**) The failure waveforms and the schematic circuit diagram of the avalanche stress system. (**b**) The waveforms adopted to stress the device.

Figure 2 expresses the schematic circuit diagram of the avalanche stress system and the oscilloscope waveforms generated by the system on the asymmetric trench SiC MOSFET. A drive circuit controlled the ON and OFF of the device under testing (DUT). The gate-source voltage (V_gs_) was set from 0 to 18 V. The DUT was connected in series with an inductor (L = 1 mH). The power supply voltage (V_DD_) was 100 V. As shown in Figure 2b, when the gate of the DUT turns ON, the drain-source current (I_ds_) gradually rises. The rising slope of the current was proportional to the V_DD_ and inversely proportional to the inductance value:

(1) $$\frac{di}{dt}=\frac{V_{DD}}{L}$$

When the gate of the DUT turns OFF, the energy stored in the inductor is dissipated on the DUT. At this time, the SiC MOSFET is under the avalanche state. The V_ds_ equals to the BV and the current flows through the inductor following the formula:

(2) $$\frac{di}{dt}=-\frac{BV-V_{DD}}{L}$$

When the current in the circuit falls to 0 A, the energy stored in the inductor is completely consumed and the avalanche state of the device ends.

When applying the avalanche stress on the device and gradually increasing the gate pulse width until it fails, the asymmetric trench SiC MOSFET can endure a peak I_load_ (I_peak_) of 22 A, as shown in Figure 2a. All the three ports of the failed device were short-circuited, indicating that the device dies from thermal runaway [25]. The avalanche waveforms with an I_peak_ of 18 A, which is 80% of 22 A, as shown in Figure 2b, were adopted here to repetitively stress the asymmetric trench SiC MOSFET. The gate pulse width was 180 μs and the I_ds_ increased to 18 A at a rate of 0.1 A/μs. After that, the gate of the device turns OFF and the device is under the avalanche state while the V_ds_ is kept at 1600 V.

**Figure 3:**
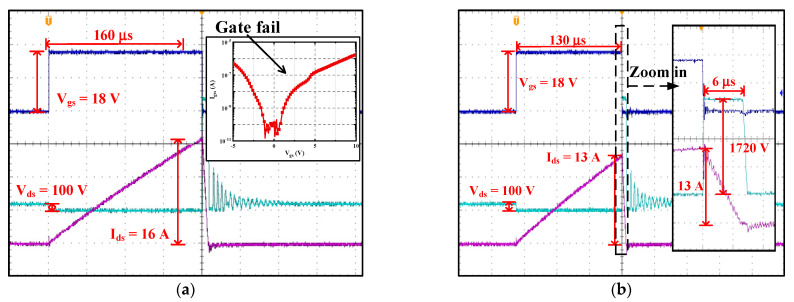
The avalanche waveforms of the double-trench SiC MOSFET. (**a**) The failure waveforms. (**b**) The waveforms adopted to stress the device.

The avalanche-induced failure waveforms of the double-trench SiC MOSFET are shown in Figure 3a. After enduring a 16 A-avalanche stress, the gate leakage current (I_gss_) is higher than 1 μA, indicating that the gate failure is the dominant mechanism [18,19]. Obviously, the asymmetric trench SiC MOSFET can endure much more serious single-pulse avalanche stress than the double-trench one, implying that the asymmetric structure has a better protection effect [25]. Similarly, the avalanche stress with 13 A I_peak_, which is 80% the maximum avalanche current the device can endure, was adopted to stress the double-trench SiC MOSFET. The stress waveforms are presented in Figure 3b. The gate pulse width was 130 μs. When under the avalanche state, the V_ds_ of the double-trench device is 1700 V.

In addition, during the repetitive avalanche stress experiments for both the devices, the duty cycle of all pulses was 0.1%. Wind heat dissipation was also added to suppress the rise in the junction temperature. The repetitive avalanche stress with extremely high voltage and current will lead to the degradation of the devices, which is going to be analyzed in Section 3.

## 3. Results and Discussions

### 3.1. Degradation of Electrical Characteristics

#### 3.1.1. Asymmetric Trench SiC MOSFET

**Figure 4:**
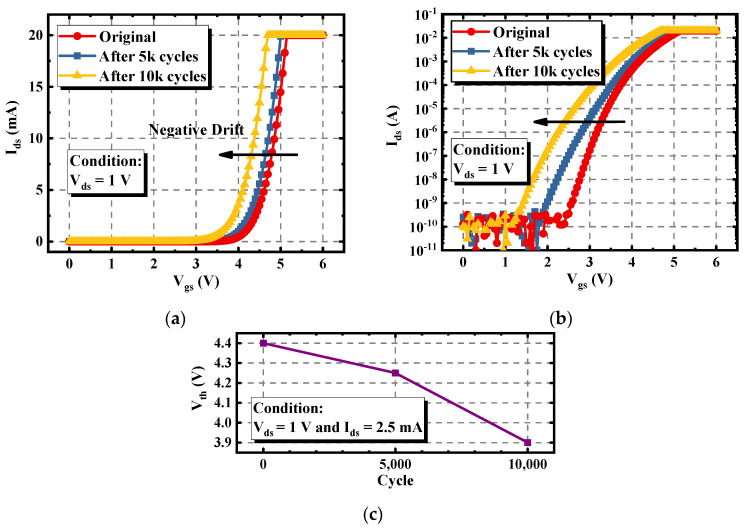
Variations of the I_d_-V_g_ characteristic of the asymmetric trench SiC MOSFET under different avalanche stress cycles. (**a**) I_d_-V_g_ curves in the linear scale. (**b**) I_d_-V_g_ curves in the logarithmic scale. (**c**) Extracted degraded V_th_.

After enduring repetitive avalanche stress, the electrical characteristics under different stress cycles were measured and compared. Figure 4 shows the degradation in the threshold voltage (V_th_) of the asymmetric trench SiC MOSFET. With the increase of the total stress cycles, the V_th_ curves shifted to the negative direction. The V_th_ under the condition of V_ds_ = 1 V and I_ds_ = 2.5 mA is extracted in Figure 4c. After enduring 10k cycles, the V_th_ of the asymmetric device dropped from 4.4 to 3.9 V. In the logarithmic scale, the V_th_ at I_ds_ = 1 nA was reduced by 1.15 V, as shown in Figure 4b, which is more obvious than that in the linear scale. This indicates that after enduring the repetitive avalanche stress, the channel of the asymmetric trench SiC MOSFET degrades.

**Figure 5:**
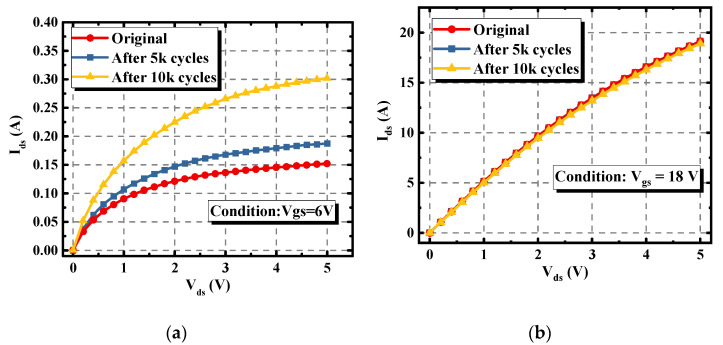
Variations of the I_d_-V_d_ characteristic of the asymmetric trench SiC MOSFET under different avalanche stress cycles. (**a**) I_d_-V_d_ curves under the V_gs_ = 6 V bias condition. (**b**) I_d_-V_d_ curves under the V_gs_ = 18 V bias condition.

The variations of the I_d_-V_d_ characteristics of the asymmetric trench device under the conditions of V_gs_ = 6 V and V_gs_ = 18 V are plotted in Figure 5a,b. The I_ds_ under the condition of V_gs_ = 6 V increased due to the decrease of the V_th_. Meanwhile, the I_d_-V_d_ curve remained stable when the device was biased under the condition of V_gs_ = 18 V. This is because the channel of the device was fully open and the channel resistance was relatively low when compared with the resistances of the junction field effect transistor (JFET) region and N-drift region. This also indicates that the JFET region and drift region of the asymmetric trench SiC MOSFET are rarely affected by the repetitive avalanche stress.

**Figure 6:**
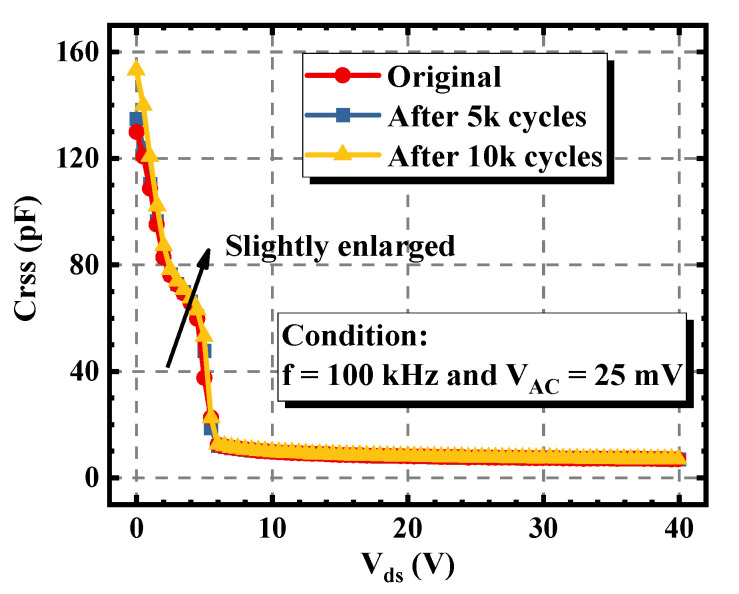
Variations of the C_gd_ of the asymmetric trench SiC MOSFET under different avalanche stress cycles.

The variations of the capacitance characteristic of the asymmetric trench device were also measured. As shown in Figure 6, with the increase of the stress cycles, the gate-drain capacitance (C_gd_) of the device increased slightly. Within 10k cycles, the maximum C_gd_ rose from 128 pF to 154 pF, equaling to an increment of 20.3%. This is because there were positive charges injected into the left corner and the bottom interface of the gate trench, which will be explained later in Section 3.2.

#### 3.1.2. Double-Trench SiC MOSFET

**Figure 7:**
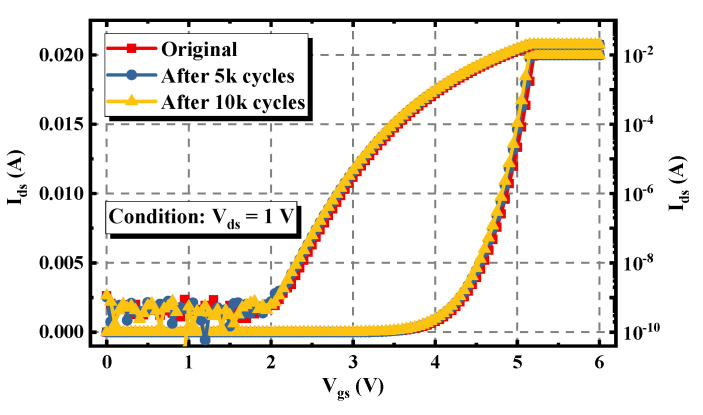
Extracted I_d_-V_g_ characteristic of the double-trench SiC MOSFET under different avalanche stress cycles.

**Figure 8:**
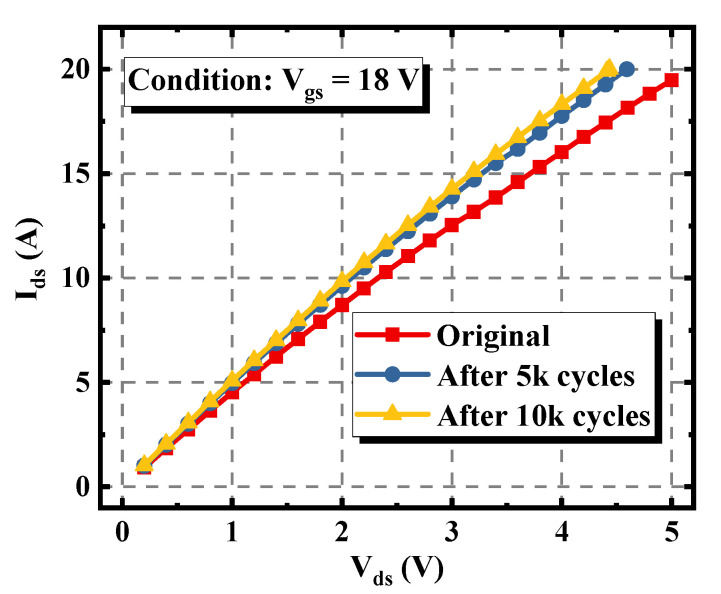
Variations of the I_d_-V_d_ characteristic at V_gs_ = 18 V of the double-trench SiC MOSFET under different avalanche stress cycles.

The repetitive avalanche experiment was also performed on the double-trench SiC MOSFET. As can be seen in Figure 7, the V_th_ of the device only expressed a slight negative shift during the experiment, implying that the channel of the device was rarely degraded, which is quite different from the phenomenon monitored in Figure 4. Meanwhile, as shown in Figure 8, with the increase of the stress cycles, the I_d_-V_d_ curve under the condition of V_gs_ = 18 V rose significantly. This means that the R_on_ at V_gs_ = 18 V and I_ds_ = 18 A was decreased by 14%.

The repetitive avalanche experiment was also performed on the double-trench SiC MOSFET. As can be seen in Figure 7, the V_th_ of the device only expressed a slight negative shift during the experiment, implying that the channel of the device was rarely degraded, which is quite different from the phenomenon monitored in Figure 4. Meanwhile, as shown in Figure 8, with the increase of the stress cycles, the I_d_-V_d_ curve under the condition of V_gs_ = 18 V rose significantly.

**Figure 9:**
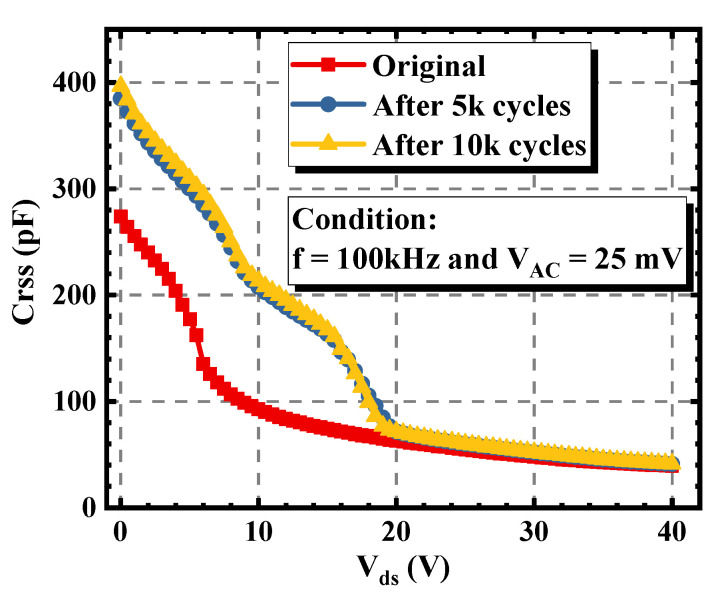
Variations of the C_gd_ of the double-trench SiC MOSFET under different avalanche stress cycles.

As plotted in Figure 9, after enduring 10k avalanche stress cycles, the maximum C_gd_ of the double-trench device increased from 274 pF to 397 pF, equaling to an increment of 44.9%. This obvious degradation resulted from the positive charges injected into the bottom oxide [20]. This indicates that compared with the asymmetric trench SiC MOSFET, much more positive charges were injected into the trench bottom oxide of the double-trench device during the avalanche process, making the C_gd_ change greatly. At the same time, the positive charges attracted electrons and decreased the resistance of the JFET region. Therefore, the V_th_ of the double-trench SiC MOSFET was unchanged while the R_on_ was reduced.

### 3.2. Simulations and Analysis

**Figure 10:**
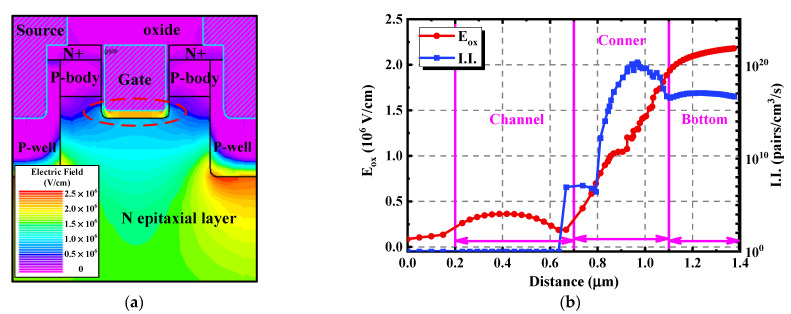
Physical characteristics of the double-trench SiC MOSFET under the avalanche state. (**a**) Distribution of the electric field. (**b**) Extracted E_ox_ and I.I. along the gate oxide interface.

Simulations were then performed to help analyze the various degradation trends between the two types of devices with different structures. Figure 10a reflects the electric field distribution of the double-trench SiC MOSFET under the avalanche state. The highest electric field was located at the bottom of the source trench, where the avalanche breakdown occurred. Meanwhile, the electric field at the gate trench cannot be ignored. The oxide electric field (E_ox_) and the impact ionization rate (I.I.) along the gate oxide interface are extracted in Figure 10b. The peak value of the E_ox_ appeared at the bottom, pointing from the semiconductor to the oxide, while the peak I.I. appeared at the corners. This illustrates that the E_ox_ and I.I. at the bottom and corners of the trench together lead to the injection of positive charges into the gate oxide, resulting in an increase in C_gd_ and decrease in the resistance of the JFET region. There was no I.I. in the channel region of the double-trench device, meaning that the channel was not affected by the stress, which is consistent with the measured data in Figure 7.

**Figure 11:**
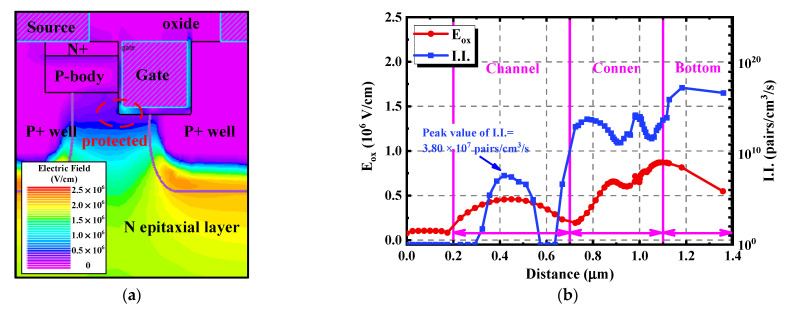
Physical characteristics of the asymmetric trench SiC MOSFET with a vertical channel under the avalanche state. (**a**) Distribution of the electric field. (**b**) Extracted E_ox_ and I.I. along the gate oxide interface.

Furthermore, an asymmetric trench SiC MOSFET structure with a vertical channel was built to help analyze the different degradations resulting from the double-trench and the asymmetric structures, ignoring the influence brought by the inclined channel. The physical characteristics of it under the avalanche state are shown in Figure 11. As can be seen in Figure 11a, the peak value of the electric field was located at the bottom of the deep P+ well. Different from the double-trench SiC MOSFET, due to the narrower JFET region, the serious E_ox_ did not appear at the bottom of the trench in the asymmetric device. Comparing Figure 11b with Figure 10b, it can be concluded that both the E_ox_ and the I.I. along the trench bottom and the corner of the asymmetric device are much lower than those in the double-trench device. The peak E_ox_ fell from 2.18 × 10^6^ V/cm to 8.51 × 10^5^ V/cm, decreasing by 61.0%. Meanwhile, the peak I.I. reduced from 1.46 × 10^20^ pairs/cm^3^/s to 1.70 × 10^17^ pairs/cm^3^/s, equating to a nearly three-orders of magnitude reduction. This demonstrates that the deep P+ well in the asymmetric trench SiC MOSFET can protect the bottom gate oxide well. This is why the double-trench SiC MOSFET shows much more serious degradation in the C_gd_ and the I_d_-V_d_ curve under the high gate bias condition. Moreover, it is worth noting that there existed a 3.80 × 10^7^ pairs/cm^3^/s I.I. peak in the channel, even though it was still minor.

**Figure 12:**
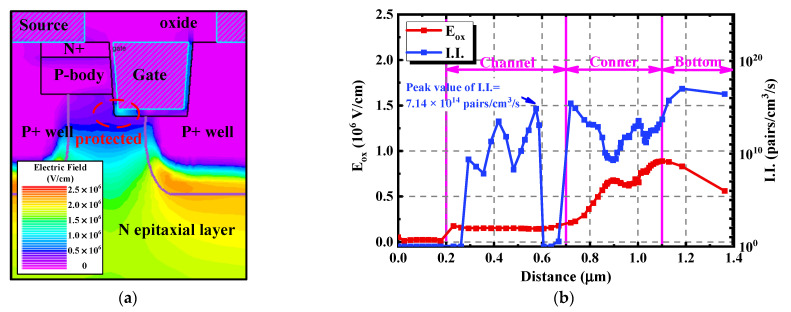
Physical characteristics of the asymmetric trench SiC MOSFET with an inclined channel under the avalanche state. (**a**) Distribution of the electric field. (**b**) Extracted E_ox_ and I.I. along the gate oxide interface.

The real asymmetric trench SiC MOSFET produced by Infineon adopted the (11–20) crystal plane to form the channel, making the channel inclined. Figure 12 simulates the physical characteristics of the device. The most obvious difference between Figure 12b and Figure 11b is that a much higher I.I., reaching a peak value of 7.14 × 10^14^ pairs/cm^3^/s, appears at the inclined channel region. This is because the inclined crystal plane made the channel become exposed to the avalanche stress, promoting the positive charges to be injected into the channel oxide. Therefore, the V_th_ of the asymmetric trench SiC MOSFET continued to decrease, just as presented in Figure 4. The simulation results are mostly agreeable with the measured data, proving the correctness of the mechanisms discovered and the investigation made in this work.

## 4. Conclusions

Based on the measured discrepant degradation trends between the double-trench SiC MOSFET and the asymmetric trench SiC MOSFET before and after enduring the repetitive avalanche stress, the mechanisms brought by the different device structures were revealed. On the one hand, since ultrahigh peak values of E_ox_ and I.I. appeared at the bottom oxide of the double-trench SiC MOSFET under the avalanche state, the positive charges injected there contributed to the obvious decrease of the R_on_ and to the increase of the C_gd_. These phenomena were not observed in the asymmetric trench device because the deep P+ well protected the bottom gate oxide well. On the other hand, the inclined channel of the asymmetric device attracted a much higher I.I. in the channel region, leading more positive charges to be injected into the oxide there. Hence, the degradation of the V_th_ of the asymmetric trench SiC MOSFET was more obvious. The differences in the degradation brought about by the different device structures were summarized and verified. Different from the double-trench SiC MOSFET, the gate oxide at the corner of the trench of the asymmetric trench SiC MOSFET device was well protected. However, when adopting inclined channel, the channel of the asymmetric device is more vulnerable to damage. Therefore, the reliability of both the bottom and the sidewall of the trench needs to be considered in the trench design. It is believed that for these two types of trench-gate SiC MOSFETs, there also exists different degradation phenomena induced by other repetitive stresses, such as the short-circuit stress, the power cycling stress, and the surge current stress, which will be studied in detail in the future.

## Figures and Tables

**Table 1:** Device electrical parameters.

Electrical Parameters	Double-Trench MOSFET (SCT3160KL)	Asymmetric Trench MOSFET (IMW120R140M1H)
R_on_ (mΩ)	160	140
I_D_ (A)	18	19
BV@I_dss_ = 1 uA (V)	1814	1479

## Data Availability

Data sharing is not applicable for this article.
